# Economic Impact of Pharmacist-Participated Medication Management for Elderly Patients in Nursing Homes: A Systematic Review

**DOI:** 10.3390/ijerph16162955

**Published:** 2019-08-16

**Authors:** Arim Kwak, Yoo Jin Moon, Yun-Kyoung Song, Hwi-Yeol Yun, Kyungim Kim

**Affiliations:** 1 College of Pharmacy, Korea University, Sejong 30019, Korea; 2 Independent Researcher, Laurel, MD 20723, USA; 3 College of Pharmacy, Daegu Catholic University, Gyeongsan-si, Gyeongbuk 38430, Korea; 4 College of Pharmacy, Chungnam National University, Daejeon 34134, Korea; 5 Biomedical Research Center, Korea University Guro Hospital, Seoul 08308, Korea

**Keywords:** systematic review, economic outcome, pharmacist, interprofessional, medication management, nursing home, elderly

## Abstract

This systematic review examined the varied studies that have assessed the economic impact of pharmacist-participated medication management for nursing home residents older than 65 years of age. The articles published during 1990–2017 were found through PubMed, EMBASE and Ovid Medline. After the selection process by independent reviewers, a total of 12 studies were included. The quality of the selected articles was assessed using the Effective Public Health Practice Project checklist for economic studies. The articles were highly heterogeneous in terms of study design, pharmacist participation type, and measures of economic outcome. Therefore, the results are presented narratively according to the type of pharmacist involvement featured in the articles: interprofessional networks, interprofessional coordination, or interprofessional teamwork. Of the eight studies performing statistical comparison analyses, one study of interprofessional coordination and three of interprofessional teamwork showed statistically significant positive economic outcomes. The remaining four studies showed non-significant tendencies towards favorable economic outcomes. This review provides insights into the essential features of successful pharmacist-participated medication management for elderly patients in nursing homes.

## 1. Introduction

Nursing home care is an important component of the care services provided for the elderly population. In many societies, as the proportion of elderly people continues to increase, the nursing home resident population also increases and ages [1]. Compared with the community-dwelling elderly, nursing home residents generally require more medication and this puts them at greater risk of inappropriate medication use [2,3]. Indeed, in nursing homes multiple medication use (polypharmacy) and inappropriate prescription are frequently reported problems [4,5,6]. While the rate of potentially inappropriate medication use is about 20% in community settings [7,8], almost half of nursing home residents are exposed to potentially inappropriate medication [9]. For these reasons, there has been a focus on the quality of medication management in this vulnerable population [10,11].

Inadequate care of the elderly is not only a safety concern but also a factor behind excessive healthcare expenditure [12]. Indeed, drug-related morbidity and mortality in nursing homes poses a great economic burden [13]. A previous study reported that the average expenditure per elderly person for nursing home residents was five times higher than for community residents, and that the overall national expenditure was three times higher [14]. As a possible response to this issue, incorporating pharmacist input into medication management has been suggested and implemented. It is well known that pharmacist involvement can improve medication management for the elderly through reducing inappropriate prescriptions [15,16]. Furthermore, in both U.S. and Europe ensuring the effective use of medication therapy and reducing the unnecessary costs caused by drug-related problems has generated huge cost-savings.

Incorporating pharmacist activities into medication management, however, demands a significant time commitment from the pharmacist and utilizes healthcare resources. Regardless of the efficacy gains, healthcare policy-makers or other stakeholders frequently require evidence supporting the cost-effectiveness of this approach [17]. Thus, given the rising healthcare costs in this era, it is important to explore the cost effectiveness of pharmacist-participated medication management in nursing homes. Yet, compared to the extensive studies carried out measuring the safety or economic impact of pharmacist participation with the community-dwelling elderly, limited effort has been applied to studying the economic outcomes in nursing homes. This leaves the economic impact of mandating pharmacist activities of various participation types in nursing homes questionable, despite their promising potential. Thus, the main purpose of this review was to examine the studies undertaken of the economic outcomes of various types of pharmacist medication management participation for nursing home residents.

## 2. Materials and Methods

### 2.1. Search Strategy

The search strategy of this study was designed to retrieve and produce an unbiased analysis of the research into the economic effects of pharmacist-participated medication management in nursing homes. Databases (PubMed, EMBASE and Ovid Medline) covering articles published in English between January 1990 and July 2017 were searched using appropriate search terms adapted for each bibliographic source. These databases were searched for the MeSH or text keywords “nursing home”, “long term care facility”, “pharmacist”, “pharmacy”, “pharmaceutical care”, “interdisciplinary”, “costs”, “cost analysis”, “economics”, and related words in different combinations. The subject headings used as search terms were split into four categories: those related to pharmacy (“pharmacy” OR “pharmacist” OR “pharmaceutical care”); AND those related to pharmacist participation (“interdisciplinary” OR “multidisciplinary”); AND those related to cost (“economics” OR “cost” OR “cost analysis”); AND those related to nursing home (“nursing home” OR “long term care” OR “aged facility”). The search terms were used in title, abstract, keywords searches. There were no restrictions regarding the study design or method of economic analysis, reflecting the need to be over-inclusive in an attempt to be complete.

### 2.2. Study Selection

All the references retrieved by the literature search were screened by two independent reviewers (the first and second authors) based on the title and abstract of each citation. The articles retrieved could be eliminated if their titles clearly indicated that they did not focus on the economic outcomes of pharmacist activities in nursing homes. Next, the remaining abstracts were examined to identify papers that measured the cost or cost-effectiveness of pharmacist-participated medication management for elderly in nursing homes. The articles to be included in the study were finalized after a full review of each paper. The inclusion criterion was whether the full-text of the articles was primarily focused on economic analysis of pharmacist participation in nursing homes. To access the full-text of the articles, the current authors utilized their institutions’ electronic resources and tried to contact the original authors via e-mail. The following types of articles were excluded from the review: those featuring home or residential nursing settings lacking care services provided by registered healthcare professionals, such as nurses on duty at all times, those that had residents under 65 years old, those that were about the nursing home system itself rather than the residents within, or those that included interventions not carried out by pharmacists (e.g., by nurses or other healthcare professionals or multidisciplinary teams without a pharmacist). The full-text review also excluded articles measuring only clinical outcomes or those based on the same study. Throughout the selection process, any disagreement was resolved either by discussion among the reviewers or by considering the opinion of additional reviewers (the corresponding author and the other two co-authors) to reach a consensus.

### 2.3. Quality Assessment

The two independent reviewers (the first and second authors) assessed the quality of the selected articles using the “Quality Assessment Tool For Quantitative Studies” developed by the Effective Public Health Practice Project (EPHPP), which, given its content and construct validity, has been judged suitable for use in systematic reviews of effectiveness [18,19]. The scoring was based on the objective guidelines provided by the EPHPP. Eight domains were assessed: selection bias, study design, confounders, blinding, the data collection method, withdrawals/dropouts, intervention integrity, and analysis. Following the guidelines of the tool, for each article, every domain except intervention integrity and analysis was rated as either strong (three points), moderate (two points), or weak (one point), and then these scores were summed to provide the total score. Based on their total score, the studies were assigned a quality rating of weak (two or more weak ratings), moderate (one weak rating), or strong (no weak ratings) for their overall methodology.

### 2.4. Data Extraction and Analysis

The two independent reviewers (the first and second authors) extracted data from each article including the study design, characteristics of the enrolled patients, type of activity, type of economic analysis, and the outcome measures of interest. Any disagreement was resolved either by discussion among the reviewers or by considering the opinion of additional reviewers (the corresponding author and other two co-authors) to reach consensus. Because of the high degree of heterogeneity in terms of study design, activity type, and economic outcome measures, a meta-analysis could not be performed. Instead, the results are presented as a narrative review. To synthesize the various participation types, the studies were categorized according to the main activity type based on the level of pharmacist participation: interprofessional networks, interprofessional coordination, or interprofessional teamwork. These terms are based on the glossaries published in previous studies [20,21]. That is, the activities were categorized as interprofessional networks if a pharmacist’s main activities were limited to attending multidisciplinary case conferencing on a periodic basis. They were categorized as interprofessional coordination if they included mainly pharmacist-led medication reviews in which pharmacists provided advice to prescribers, yet left the implementation of such advice to other practitioners. Finally, they were considered interprofessional teamwork if they were cases of interventions through regular team care in which activities to solve problems and deliver services were carried out collaboratively and in an integrated and interdependent manner rather than being solely pharmacist-driven. Note that the monetary values in the tables are presented as they were in the original papers.

## 3. Results

### 3.1. Search Results

**Figure 1:**
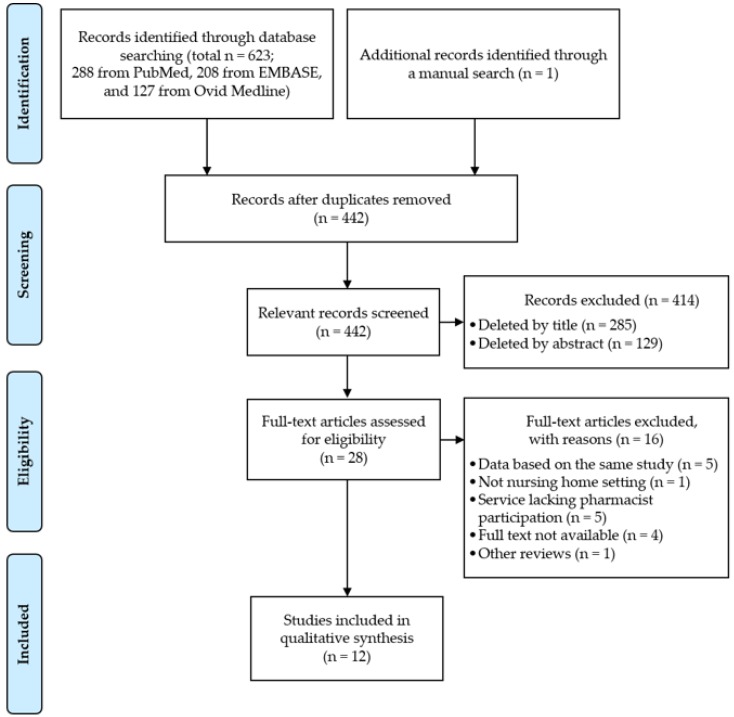
Study selection process.

Figure 1 presents the flow chart of the literature search process as suggested by PRISMA [22]. The initial number of articles found was 288 from PubMed, 208 from EMBASE, and 127 from Ovid Medline (623 in total). One further article was identified through a manual search of the reference lists of the selected articles. After the removal of 182 duplicates, 285, 129, and 16 studies incompatible with our inclusion criteria were excluded at the title, abstract, and full-text levels, respectively. The most common reasons for the exclusion of articles throughout the selection process were that they did not evaluate economic outcomes or focus on nursing home residents. Of the 28 articles included in the full-text review, 12 were deemed eligible for inclusion in the systematic review.

### 3.2. Quality Assessment

Each study’s quality was evaluated using the EPHPP guidelines (Table 1). According to the guidelines of the tool, five studies were rated of strong quality, four studies were rated of medium quality, and the remaining three were considered as weak quality. The most common reasons for a low-quality grading were a lack of controlled confounding factors and the incomplete follow-up of residents. In many of the randomized studies, little information on the allocation was available.

### 3.3. Literature Evaluation

Of the 12 articles, two studies were in the context of interprofessional networks [25,28], six in interprofessional coordination [23,24,26,29,33,34], and four in interprofessional teamwork [27,30,31,32]. The analytic methods of the cost outcomes varied; there were seven cost-minimization analyses [24,25,26,27,28,31,34], two cost–benefit analyses [23,29], two cost-effectiveness analyses [30,32], and one cost–utility analysis [33]. Regardless of the outcome type, the outcomes of all the studies were positive in direction or favorable.

#### 3.3.1. Effect of Interprofessional Networks

The two studies involving interprofessional networks both resulted in non-significant but favorable economic outcomes (Table 2) [25,28]. In King and Roberts (2001), weekly multidisciplinary case conferences were held on cases selected by general practitioners in three nursing homes [25]. In a conference, three general practitioners, one clinical pharmacist, and other staff and healthcare professionals discussed and agreed on a case management plan. The pharmacist reviewed and prepared notes on medication and related problems to be presented, with the support of a general practitioner’s physical examination and a medical record review. The intervention resulted in 170 recommendations suggested by the pharmacist (*n* = 102) or the committee (*n* = 68), 158 (93%) of which were drug-related recommendations. Of those recommendations, 92 were implemented. When the median changes in weekly medication cost over the study period were calculated, there was a reduction in the intervention group, but not in the control group (Australian dollar (AUD) −0.16 in the intervention group vs. AUD 0 in the control group, *p* = 0.75). The authors reported the calculated saving due to multidisciplinary case conferences as being approximately AUD 154 per review, which was remarkably similar to the maximum AUD 153 cost paid per review to cover pharmacist and general practitioner time.

The study by Crotty et al. (2004) focused on an outreach advisory service of multidisciplinary case conference reviews [28]. Two multidisciplinary case conferences were conducted 6–12 weeks apart in which the resident’s general practitioner, a geriatrician, a pharmacist and residential care staff participated. A problem list covering the selected residents with medication problems and/or challenging behaviors (*n* = 154) was developed by the general practitioner, and medication reviews were conducted before each case conference. Then, the monthly drug costs for all regular medications on the government’s Pharmaceutical Benefits Scheme schedule were calculated. The mean change in the estimated total monthly drug cost was AUD 5.72 in the intervention group and AUD 3.37 in the control group (*p* = 0.837). In this study, the authors mentioned that multidisciplinary case conferences in nursing homes could improve care but that the major obstacle was the time required to organize a meeting and the challenge of coordinating a group of multidisciplinary health professionals.

#### 3.3.2. Effect of Interprofessional Coordination

Six studies investigated the economic impact of interprofessional coordination, which included pharmacist-led medication reviews of nursing home residents (Table 3) [23,24,26,29,33,34]. There were two observational cohorts [23,29], two cluster randomized controlled trials (RCTs) [24,26], one controlled clinical trial [33], and one before-and-after study [34].

The studies by Cooper (1997, 2007) both included cohorts of residents in one nursing home [23,29]. A consultant pharmacist evaluated problem-oriented drug regimen reviews and assessments on a monthly basis. The medication reviews mostly concerned adverse drug reactions and interactions, medication discontinuation, and psychotropic conversion. Cooper (1997, 2007) calculated the cost/benefit savings realized by the pharmacists’ recommendations accepted by physicians and the potential savings lost through rejection. He highlighted the cost saving disparity resulting from the difference between those recommendations accepted and those rejected by the physicians. In this study, the cost–benefit analysis used the ratio of the costs of the drugs, lab tests, drug administration labor, and hospitalization outcomes to the cost of the consultant pharmacist services. The cost of providing consultant pharmacist services was determined via a capitation per patient-month fee and travel over the year. In an earlier study (1997), the first two years of intervention resulted in a cost/benefit ratio of US dollar (USD) 10.2/1 from the recommendations accepted and USD 10.3/1 unrealized due to rejection by physicians. Thus, the total cost/benefit ratio if all the recommendations had been accepted would have been USD 20.5/1 [23]. In a study of 2007, the results of the fourth year were a realized cost/benefit ratio of USD 10.5/1 from those recommendations accepted and a potential further USD 20.9/1 from those rejected leading to a total potential cost/benefit ratio of USD 31.4/1 [29].

A randomized controlled trial by Burns et al. (2000) evaluated the cost impact of medication reviews by calculating the costs of primary and secondary care resources in 14 nursing homes over an 8-month period, with two 4-month phases: an observation phase and an intervention phase [24]. Nursing homes in the intervention group received a single medication review multidisciplinary team intervention including a pharmacist while the nursing homes of the control group did not. For the intervention group over the 4-month period, the total per resident cost associated with primary and secondary resource use, £178 (USD 284.80), was significantly reduced (*p* = 0.028) by approximately £22 (USD 35.20) in terms of medicine budget, even after accounting for pharmacist time. The cost of pharmacist time in the study was calculated per hour from national salary rates. All costs were in 1997 prices. Consistency of data collection was ensured by using a protocol and a standard form; however, no additional analysis was performed to account for the baseline discrepancy. It was noted that the study’s short timeline was a limitation and that further research was necessary to establish how long the savings would be maintained if the intervention was extended over longer periods.

Roberts et al. (2001) examined the impact of a clinical pharmacy service model of medication reviews for one year, supported by nurse education but without any direct contact with physicians [26]. Drug use in the intervention group was reduced by 14.8% compared to that of the control group. A net cost saving was AUD 16 per resident with a projected total annual saving of AUD 1.2 million across Australia’s nursing home population. This was a large-scale cluster RCT designed study and applied a stratified random sampling strategy. In addition, clear eligibility criteria and pharmacist qualifications were established, and the intervention comprised multiple components such as focus groups and the use of valid claims data. However, as the authors reported, the low acceptance rate by physicians (39%) was a limitation.

The study by Jõdar-Sánchez et al. (2014) analyzed the cost/utility value of the studied intervention by considering the differences between groups’ costs and quality adjusted life years (QALYs) [33]. The intervention was a pharmacist’s pharmacotherapy follow-up that included an evaluation based on information drawn from interviews and historical reviews, followed by an action plan proposed to the participant and general practitioner when the negative outcomes associated with the medications were detected. The study took account of direct costs such as the pharmacist interventions and the prescribed medication costs. Costs were presented as euros and USD in 2013 prices and exchange rates. The authors recognized the risk of bias related to the nonrandom selection of nursing home residents included in the study and thus presented incremental cost effectiveness ratios (ICERs) in three scenarios to minimize this risk: cost unadjusted, cost adjusted for baseline, and cost adjusted for more group differences. When the willingness to pay was USD 38,487/QALY, the threshold for determining whether a health technology is cost effective in Spain, the intervention remained cost effective in the second and third scenarios despite larger uncertainties.

Chia et al. (2015) was the only study of an Asian population included [34]. This was a retrospective period prevalence study with two distinct phases: a one-month pre-setup period and a six-month post-setup period. During the pre-setup period, a monthly medication review was conducted, while, in the post-setup period, a weekly medication review was carried out for six months. In the post-setup period, the pharmacists approached the physicians in a variety of ways depending on the preference of the medical team at each nursing home. The drug costs were calculated in Singapore dollars (SGD) using private rates, and the monthly costs of the drugs discontinued or substituted as a result of the pharmacist review were calculated as the monthly cost saving. The results showed that the interventions of a weekly medication review during the post-setup period led to decreased pharmacotherapy problems and increased cost savings and that the cumulative acceptance rate increased significantly between these periods (*p* < 0.0001). Both total direct cost savings and mean cost savings per recommendation were higher during the post-setup period than during the pre-setup period. However, the net savings, which factored in the costs of the pharmacist review (SGD 60 per hour) could only be recovered by the direct cost savings of the post-setup period (a net saving of SGD 76.69 per month). Despite the limitation of considering only direct cost savings, the study revealed that frequent pharmacist medication reviews can boost the acceptance rate of pharmacists’ recommendations and ultimately lead to cost savings.

#### 3.3.3. Effect of Interprofessional Teamwork

Four studies evaluated the effect of interprofessional teamwork-based pharmacist participation in the medication management of nursing home residents [27,30,31,32], and three studies showed statistically significant findings regarding the economic outcomes [27,30,31] (Table 4).

Christensen et al. (2004) presented the results of the first phase of the North Carolina polypharmacy initiative, a state-wide program involving pharmacists, physicians, and local communities [27]. During the study, retrospective drug regimen reviews were performed targeting residents at high risk of drug-related problems or polypharmacy (over 18 prescription fills in the previous 90-day period), focusing on residents with the potential for cost savings. Pharmacists were introduced to the program and toolkit criteria through meetings, conference calls, and training to ensure the interventions were consistent. This resulted in a mean reduction in the number of prescriptions per month (0.21) and a mean reduction in monthly drug costs (USD 30.33 per resident, *p* < 0.001). Allowing for the cost of pharmacist payments (USD 12.50) and the combined administrative costs of the program revealed a significant saving in drug costs (cost minimization ratio = 12:1). All costs were in 2002 USD. However, despite the significant findings, the authors reported that several limitations remained due to the study being of a before-after design using a targeted population with a high prescription fill rate.

Vu et al. (2007) investigated the cost effectiveness of wound care by teams of trained pharmacists and nurses [30]. This study proved that trained pharmacist involvement in a wound care team in the intervention group was a core intervention element and resulted in significantly lower mean treatment costs than the control group which received usual care from nurses. All costs were in AUD at 2000 prices. The incremental net benefit was a reduction (unadjusted) in mean treatment costs of AUD 357.7 (*p* = 0.006) after including training costs, with an adjusted predicted cost saving per wound of AUD 277.9. The estimated correlation between the costs and the rates of wound healing was negative, and the net benefits were always positive for any non-negative social willingness-to-pay for a day without a chronic wound. In other words, the intervention resulted in both significant cost savings and significantly improved outcomes. The strengths of the study included incorporating numerous nursing homes and delineating most processes and diverse cost measurements. In addition, most covariates were controlled for using statistical methods. However, the influence of wound state heterogeneity remained a confounding factor.

The intervention examined by Locca et al. (2009) was a service newly introduced to Swiss nursing homes called the Pharmaceutical Care Service, which aimed to promote rational drug use for elderly residents through networking among doctors, pharmacists, nurses, and administrative directors [31]. This study included 22 pharmacists in 42 nursing homes, and assessed the economic impact of the service since its start in 2002 with retrospective analysis over an 8-year period (1998–2005). The study focused on the implementation of the service and prepared a systematic program to facilitate it. Interprofessional teamwork was considered a core element because of the presence of interdisciplinary training courses and symposia, discussion meetings, and educational programs for pharmacists, physicians, and nurses. After the service was implemented, the annual drug cost per resident decreased significantly (*p* = 0.005). As mentioned by the authors, the results of this study highlighted the importance of innovative pharmaceutical care services designed to assist pharmacists in the implementation of changes of practice.

Patterson et al. (2011) evaluated the cost effectiveness of an adapted U.S. model of pharmaceutical care performed by pharmacists trained to provide consistent care delivery [32]. The so-called Northern Island model was a monthly series of pharmaceutical care procedures comprising assessments of pharmaceutical care needs and medication-related problems and recommendations supported by general practitioner consultations and nursing home visits. The pharmacists reviewed residents’ medications to optimize psychoactive prescribing and made recommendations after discussions with staff and meetings with general practitioners. Despite the non-significant difference in the mean costs of the healthcare resources used, there was a negative ICER when calculated in 2005 USD, indicating that residents in the intervention group had lower costs and better outcomes than those in the control group. To be precise, the intervention group had a USD 130.39 lower cost per resident and a 30.9 percentage point reduction in the proportion of residents receiving one or more inappropriate psychoactive drugs. This study also used a cost effectiveness acceptability curve (CEAC) to highlight the low uncertainty of its results. The CEAC indicated that the probability of the service being more cost-effective than usual care was 60% even at a threshold of USD 0, which a decision-maker would be willing to pay to avoid a resident receiving one or more inappropriate psychoactive medications. However, the study was conducted on a per-protocol analysis, and thus included only 75% of all residents with complete cost and outcome data available for the intervention period.

## 4. Discussion

As the elderly population increases globally, in many developed countries the number of elderly who require long-term care services in nursing homes as well as the prices of their medicines have been steadily increasing. The rising health care expenditure of this age group is placing upward pressure not only on per capita expenditures but also on total national medical expenses [35]. This review summarizes the studies that measured the economic value of pharmacist participation in the medication management of the elderly living in nursing homes, and identifies a body of evidence indicating positive results in terms of cost-related outcomes. Recent systematic reviews have evaluated the economic outcomes of pharmacist interventions for community-dwelling elderly [36,37]. Others have evaluated care facilities for the aged including not only nursing homes but also the residential care or mixed care homes that do not provide round-the-clock care services from registered healthcare professionals such as nurses [38]. However, to our best knowledge, this study is the first systematic review of the economic impact of pharmacist participation focused on nursing homes, that is, facilities licensed to provide personal care and skilled nursing care to residents on a 24-h-a-day basis.

In this review, the included studies were categorized into three groups according to the level of pharmacist participation. All the articles, regardless of the level of pharmacist participation studied, reported that the pharmacist participation in medication management led to positive outcomes for medication or treatment cost per resident. Of the eight studies performing statistical comparison analyses [24,25,27,28,30,31,32,34], one study of interprofessional coordination [24] and three of interprofessional teamwork [27,30,31] reported their statistical significance. It is notable that the two studies of interprofessional networks showed only non-significant outcomes despite both being rated of strong methodologic quality according to the EPHPP checklist while the studies of interprofessional coordination or interprofessional teamwork did show significant outcomes in economic terms. Yet, the studies of interprofessional networks in our review had limitations; particularly, the study durations were too short for the teams to reach peak performance and prove maximal effects. In addition, the limited sample size and risk of selection bias arising from how the cases were selected by facility staff may have affected the non-significant outcome. Thus, it would be inappropriate to conclude that the interprofessional network level of pharmacist participation is ineffective, and more correct to say that this level has the potential to prove cost-effective if the elements of appropriate study design are fulfilled.

The results of this review deserve attention because previous studies have shown that pharmacists working in isolation and in a fragmented manner have limited impact on cost effectiveness [39]. Previous studies have reported that the essential elements for effective multidisciplinary care are regular conferences [40], the participation of all professionals with increased interaction [41], and the active engagement and incorporation of different views when making patient care decisions [42]. In this regard, from this review, we can consider the essential prerequisites for pharmacist participation in medication management leading to economic benefit to be activities with structured models, communication, and sufficient time for building working relationships.

The key findings of the review follow. First, team care is facilitated by a structured model with shared goals and clear roles. The studies with significant positive economic outcomes in this review were based on models such as a polypharmacy initiative as a collaborative program [27] and a wound treatment program [30]. Despite including different content and personnel, the models specified the role of each healthcare provider involved in service delivery as well as guidelines for the shared goals and missions of the overall team care service. Other studies have similarly suggested the importance of sharing common goals and clarifying roles to promote successful collaboration [43,44]. Second, the team members involved in the collaboration should have high levels of communication. Through this review, we found that cost-effective interventions offered interdisciplinary training and education that aimed to improve communication. Direct communication such as face-to-face and phone interaction is useful for identifying drug-related problems and improving physicians’ acceptance rates of pharmacist recommendations [43,45]. Effective communication can also improve working and personal relationships [46]. By contrast, the impact of pharmacist activities may be minimal when communication and/or good working relationships are absent [47]. Third, team members need sufficient time to get to know each other to build trust and professional respect [43]. This finding is further supported by McDonough and Doucette’s five-stage collaborative working relationships model, which is a framework that helps pharmacists develop working relationships with physicians through a series of stages [48]. In this model, the pre-relationship stage (stage 0) starts with professional awareness, after which pharmacists progress to professional recognition (stage 1). Stage 2 is exploration and trial, stage 3 is the expansion of professional relationships, and stage 4 is commitment to the collaboration. Since this model is composed of communication opportunities, sufficient time to take a step-wise approach, and the establishment of clearly defined roles and shared responsibilities, it can be applied practically to reduce costs.

Nevertheless, the findings of this study should be interpreted cautiously due to limitations. When the economic components of the studies were reviewed, few articles met the ideal requirements of the cost analysis presented in Zermansky and Silcock [49]: a cost-benefit or cost-utility analysis over a period of at least one year from a societal perspective. With respect to the economic components, only a few studies met the ideal type of economic evaluation, namely a cost-benefit or a cost-utility analysis. Cost-benefit analyses place monetary values on both inputs (costs) and outcomes, thereby allowing a comparison of interventions across the entire economy. Unlike alternative economic evaluation models, this analytic method can indicate the desirability of an intervention independent of a comparison to alternatives. Cost-utility analyses use a non-financial common metric that allows comparisons across the health sector, such as QALY, and thus can compare different drugs or technologies. Instead, most studies presented cost savings by comparing the costs incurred with those saved, which might inadequately show the quality of patient care or fail to highlight better resource use by pharmacists. With respect to the timeline of the intervention, the durations of six studies in this review were shorter than the one year requirement suggested by Zermansky and Silcock [49], and therefore may have underestimated the economic outcomes.

## 5. Conclusions

Although it is difficult to draw a firm conclusion because of the heterogeneity of the cost-related outcomes measured, this review provides not only evidence that pharmacist-participated medication management in nursing homes can help reduce medication costs, but also insights into the features of inter-professional team care essential for optimizing the benefits. However, the articles presented in the current review are subject to a range of potentially critical limitations, so further large-scale, long-term, controlled, and well-designed economic studies are necessary to support the importance of pharmacist-involved programs and clarify the essential elements of such programs.

## Figures and Tables

**Table 1:** Quality assessment of the selected articles according to the Effective Public Health Practice Project (EPHPP) guidelines.

Study (Year)	Selection Bias	Study Design	Confounders	Blinding	Data Collection Methods	Withdrawals and Drop-Outs	Intervention Integrity			Analyses				Global Rating
							% of Participants Receiving the Allocated Intervention	Intervention Consistency Measured	Unintended Intervention	Indication of the Allocation Unit	Indication of the Analysis Unit	Appropriate Statistical Methods	Analysis by Intervention Allocation Status	
Cooper et al. (1997) [23]	Moderate	Moderate	Weak	Moderate	Strong	Weak	Uncertain	Yes	No	Organization/institution	Individual	Yes	Uncertain	Weak
Burns et al. (2000) [24]	Moderate	Strong	Weak	Moderate	Strong	Strong	80–100	Yes	No	Organization/institution	Individual	Yes	No	Moderate
King et al. (2001) [25]	Strong	Strong	Strong	Moderate	Strong	Strong	<60	No	No	Individual	Individual	Yes	Yes	Strong
Roberts et al. (2001) [26]	Weak	Strong	Strong	Moderate	Strong	Strong	80–100	Yes	No	Organization/institution	Organization/institution	Yes	Yes	Moderate
Christensen et al. (2004) [27]	Strong	Moderate	Strong	Moderate	Strong	Moderate	80–100	Yes	No	Organization/institution	Individual	Yes	No	Strong
Crotty et al. (2004) [28]	Moderate	Strong	Strong	Strong	Strong	Moderate	80–100	Yes	No	Organization/institution	Organization/institution	Yes	Yes	Strong
Cooper et al. (2007) [29]	Moderate	Moderate	Weak	Moderate	Strong	Weak	Uncertain	Yes	No	Organization/institution	Individual	Yes	Uncertain	Weak
Vu et al. (2007) [30]	Moderate	Strong	Moderate	Moderate	Strong	Weak	80–100	Yes	No	Organization/institution	Individual	Yes	Yes	Moderate
Locca et al. (2009) [31]	Moderate	Moderate	Strong	Moderate	Strong	Strong	80–100	Yes	No	Organization/institution	Organization/institution	Yes	Yes	Strong
Patterson et al. (2011) [32]	Moderate	Strong	Strong	Strong	Strong	Moderate	80–100	Yes	No	Organization/institution	Individual	Yes	Yes	Strong
Jodar-Sanchez et al. (2014) [33]	Strong	Strong	Weak	Moderate	Strong	Strong	80–100	Uncertain	No	Organization/institution	Individual	Yes	Uncertain	Moderate
Chia et al. (2015) [34]	Moderate	Moderate	Weak	Moderate	Strong	Weak	80–100	Uncertain	No	Organization/institution	Individual	Yes	Uncertain	Weak

**Table 2:** Studies of interprofessional networks.

Study (Year, Country)	Type of Study	No. of NHs (Subjects)	Subject Mean Age (Years)	Duration of Intervention (Months)	Type of Economic Analysis	Main Interventions	Economic Outcomes
King et al. (2001, Australia) [25]	Controlled clinical trial	3 (245)	81.0	9	CMA	Three multidisciplinary case conference reviews by a multidisciplinary team in intervention group vs. Usual care in control group	• Median change in weekly medication cost (*p* = 0.75) = AUD (–) 0.16 for intervention vs. 0 for control
Crotty et al. (2004, Australia) [28]	Cluster RCT	10 (154)	85.0	3	CMA	Two multidisciplinary case conferences (6 to 12 weeks apart) and medication review (before each conference) in intervention group vs. Usual care in control group	• Mean change in monthly medication cost (*p* = 0.837) = AUD 5.72 for intervention vs. 3.37 for control

AUD = Australian dollar; CMA = cost-minimization analysis; NH = nursing home; RCT = randomized controlled trial.

**Table 3:** Studies of interprofessional coordination.

Study (Year, Country)	Type of Study	No. of NHs (Subjects)	Subject Mean Age (Years)	Duration of Intervention (Months)	Type of Economic Analysis	Main Interventions	Economic Outcomes
Cooper Jr (1997, USA) [23]	Prospective cohort study	1 (204)	83.2	24	CBA	Monthly patient assessment and problem-oriented medication review and recommendation by a consultant pharmacist	• For recommendations accepted = Cost saving/resident USD 1094 = Cost/benefit ratio USD 10.2/1 • If all recommendations had been accepted = Cost saving USD 447,811 = Cost/benefit ratio USD 20.5/1
Burns et al. (2000, UK) [24]	Cluster RCT	14 (335)	83.5	4	CMA	A single medication review by multidisciplinary team including pharmacist vs. Usual care in control group	• Mean medication cost/resident over the intervention phase (*p* < 0.05) = £131.54 (USD 210.46) for intervention vs. £141.24 (USD 225.98) for control • Total cost of healthcare resources used/resident over the intervention phase (*p* < 0.05) = £309.52 (USD 495.23) for intervention vs. £492.97 (USD 788.75) for control
Roberts et al. (2001, Australia) [26]	Cluster RCT	52 (3230)	N/A	12	CMA	Pharmacist medication review and recommendation, audited by geriatricians, and nurse education sessions in intervention group vs. Usual care in control group	• Estimated annual net cost saving/resident = AUD 16 • Projected annual net saving = AUD 1.2 million
Cooper Jr JW. (2007, USA) [29]	Prospective cohort study	1 (184)	84.3	12	CBA	Monthly patient assessment and problem-oriented medication review and recommendation by a consultant pharmacist	• For recommendations accepted = Cost saving/resident USD 619 = Cost/benefit ratio USD 10.5/1 • If all recommendations had been accepted = Cost saving USD 340,465 = Cost/benefit ratio USD 31.4/1
Jõdar-Sánchez F, et al. (2014, Spain) [33]	Controlled clinical trial	15 (332)	81.6	12	CUA	Medication review and recommendation about pharmacotherapy after the evaluation of the negative outcomes associated with medication based on a historical review and interview in intervention group vs. Usual care without pharmacist intervention in control group	• Daily mean medication cost change/resident = (–) €0.18 (USD 0.23) for intervention vs. (+) €0.58 (USD 0.75) for control • Adjusted ICERs = €3899/QALY (USD 5002/QALY) for the second scenario (costs adjusted for baseline drug use and QALYs adjusted for baseline utility score) = €6574/QALY (USD 8433/QALY) for the third scenario (costs and QALYs adjusted for baseline characteristics) • Probabilities of being cost effective for a WTP of €30,000/QALY (USD 38,487/QALY) = 35% for the first scenario = 78% for the second scenario = 76% for the third scenario
Chia HS, et al. (2015, Singapore) [34]	Retrospective before-and-after study	3 (480)	N/A	6	CMA	One month of intensive pharmacist review for each resident (pre-setup period) vs. Six months of weekly pharmacist reviews to ensure that every resident is reviewed once (post-setup period)	• Total direct cost savings during each period = SGD 388.30 (pre-setup) vs. 876.69 (post-setup) • Mean cost saved/recommendation (*p* = 0.39) = SGD 12.94 (pre-setup) vs. 19.06 (post-setup)

AUD = Australian dollar; CBA = cost–benefit analysis; CEA = cost-effectiveness analysis; CMA = cost-minimization analysis; CUA = cost–utility analysis; GP = general practitioner; ICER = incremental cost–effectiveness ratio; N/A = not assessed; NH = nursing home; RCT = randomized controlled trial; SGD = Singapore dollar; USD = US dollar; QALY = quality-adjusted life-years; WTP = willingness-to-pay.

**Table 4:** Studies of interprofessional teamwork.

Study (Year, Country)	Type of Study	No. of NHs (Subjects)	Subject Mean Age (Years)	Duration of Intervention (Months)	Type of Economic Analysis	Main Interventions	Economic Outcomes
Christensen et al. (2004, USA) [27]	Retrospective, before-and-after study	235 (9280)	76.8	6	CMA	• The North Carolina polypharmacy initiative, a collaborative demonstration program with multidisciplinary partners - developed action plan and proprietary toolkit for consulting pharmacists - pharmacists’ medication review, recommendation and consultation with physicians	• Mean monthly medication cost/resident (*p* < 0.001) = USD 502.96 (before intervention) vs. 472.63 (after intervention) • Cost-minimization ratio = 12:1
Vu et al. (2007, Australia) [30]	Cluster RCT	44 (176)	83.3	6	CEA	• Standardized treatment program in intervention group - a multidisciplinary wound care team - training program - treatment protocol vs. • Usual care from nurses in control group - no pharmacist involved - no wound care training program - no treatment protocol	• Mean treatment cost including training (*p* = 0.006) = AUD 616.4 for intervention vs. 977.9 for control • Adjusted estimates of cost saved/wound = AUD 277.9 (95% CI 21.6–534.1)
Locca et al. (2009, Switzerland) [31]	Prospective cohort study	42 (2214)	83.2	48	CMA	• Pharmaceutical care service (PCS) - coaching and training: working sessions, interdisciplinary discussion meetings, courses, and symposia - monitoring: an annual report to monitor the service - research: evaluations of the service	• Annual change in medication cost/resident between 2002 and 2005 (*p* = 0.005) = (+) 28.2% (without PCS) vs. (–) 16.4% (with PCS)
Patterson et al. (2011, UK) [32]	Cluster RCT	22 (253)	82.4	12	CEA	• Fleetwood Northern Island model in intervention group - pharmacist medication review and recommendation - application of an algorithm - interdisciplinary liaison vs. • Usual care without pharmacist intervention in control group	• Annual mean cost of healthcare resources used/resident (*p* = 0.80) = USD 4923 for intervention vs. 5053 for control • ICER = (–) 130.39/0.309

AUD = Australian dollar; CHF = Swiss franc; CEA = cost-effectiveness analysis; CMA = cost-minimization analysis; ICER = incremental cost–effectiveness ratio; NH = nursing home; RCT = randomized controlled trial.
